# Superinfection by discordant subtypes of HIV-1 does not enhance the neutralizing antibody response against autologous virus

**DOI:** 10.1186/1742-4690-9-S2-P72

**Published:** 2012-09-13

**Authors:** LM Mayr, RL Powell, JN Ngai, A Nádas, PN Nyambi

**Affiliations:** 1 NYU School of Medicine, New York, USA; 2 Medical Diagnostic Center, Yaounde, Cameroon; 3 Institute of Environmental Medicine, NYU School of Medicine, USA; 4 Veterans Affairs New York Harbor Healthcare Systems, New York, USA

## Background

Recent studies have demonstrated that both the potency and breadth of the humoral anti-HIV-1 immune response in generating neutralizing antibodies (nAbs) against heterologous viruses are significantly enhanced after superinfection by discordant HIV-1 subtypes, suggesting that repeated exposure of the immune system to highly diverse HIV-1 antigens can significantly improve anti-HIV-1 immunity. We investigated whether sequential plasma from subjects superinfected with discordant HIV-1 subtypes, who exhibit broad nAbs against heterologous viruses, also neutralize either or both of their discordant early autologous viruses with increasing potency.

## Methods

Sequential blood samples were collected from superinfected and singly infected subjects in Cameroon. RNA was extracted; env was amplified by nested RT-PCR and cloned in env-expression vectors. Purified vector was used to co-transfect 293T cells with the Q23-delta-env HIV-1 backbone vector. The pseudovirus supernatant was tested in neutralization assays with TZM-bl cells using patient plasma samples before and after superinfection.

## Results

Comparing the neutralization capacities of sequential plasma obtained before and after superinfection of 4 subjects to those of matched plasma obtained from 4 singly infected control subjects, no difference in the increase in neutralization capacity was observed (p=0.328). Overall, neutralization increased over time in 3 of the 4 singly infected patients (mean change in IC_50_ titer from first to last plasma sample: 183.4) and in 3 of the 4 superinfected subjects (mean change in IC_50_ titer from first to last plasma sample: 66.5). Analysis of the Breadth-Potency Scores confirmed that there was no significant difference in the increase in superinfected and singly infected study subjects (p=0.234).

## Conclusion

These studies suggest that while superinfection by discordant subtypes induces antibodies with enhanced neutralizing breadth and potency against heterologous viruses, the potency to neutralize their autologous viruses is not better than those seen in singly infected patients.

